# When Type 2 Processing Misfires: The Indiscriminate Use of Statistical Thinking about Reasoning Problems

**DOI:** 10.3390/jintelligence10040109

**Published:** 2022-11-17

**Authors:** Mário B. Ferreira, Jerônimo C. Soro, Joana Reis, André Mata, Valerie A. Thompson

**Affiliations:** 1 CICPSI, Faculdade de Psicologia, Universidade de Lisboa, 1649-013 Lisboa, Portugal; 2 Digital Human-Environment Interaction Labs, Lusófona University, HEI-Lab, 1749-024 Lisboa, Portugal; 3 Department of Psychology, University of Saskatchewan, 9 Campus Drive, Saskatoon, SK S7N 5A5, Canada

**Keywords:** dual-process theory, reasoning, judgment, bias, metacognition

## Abstract

Research on dual-process theories of judgment makes abundant use of reasoning problems that present a conflict between Type 1 intuitive responses and Type 2 rule-based responses. However, in many of these reasoning tasks, there is no way to discriminate between the adequate and inadequate use of rules based on logical or probabilistic principles. To experimentally discriminate between the two, we developed a new set of problems: rule-inadequate versions of standard base-rate problems (where base rates are made irrelevant). Across four experiments, we observed conflict sensitivity (measured in terms of response latencies and response confidence) in responses to standard base-rate problems but also in responses to rule-inadequate versions of these problems. This failure to discriminate between real and merely apparent (or spurious) conflict suggests that participants often misuse statistical information and draw conclusions based on irrelevant base rates. We conclude that inferring the sound use of statistical rules from normatively correct responses to standard conflict problems may be unwarranted when this kind of reasoning bias is not controlled for.

## 1. Introduction

One of the pioneering contributions of the initial work of the research program on heuristics and biases (Tversky and Kahneman 1974, 1983; Kahneman et al. 1982) was the careful development of several reasoning tasks that were used to demonstrate that, more often than not, people’s judgments seem to violate basic principles of probability and logic. One of these tasks was the so-called “lawyer-engineer problem” (Kahneman and Tversky 1972):

*In a study, several psychologists interviewed a group of people. The group included 5 engineers and 995^1^ lawyers. The psychologists prepared a brief summary of their impression of each interviewee. The following description was drawn randomly from the set of descriptions: Dan is 45. He is conservative, careful, and ambitious. He shows no interest in political issues and spends most of his free time on his many hobbies, which include carpentry, sailing, and mathematical puzzles. Which of the following is more likely?*

* (a) Dan is an engineer*

* (b) Dan is a lawyer*

When facing the problem above, many people might rely on the similarity between Dan’s description and the stereotype of engineer to infer that Dan is an engineer (without taking into account the prior odds of being an engineer or a lawyer; 5/995). In other words, people’s judgments often rely on intuitive processes (e.g., judgment by representativeness; Kahneman and Tversky 1972) rather than analytic ones (reasoning considering the initial base rates).

In accordance with dual-process theories of reasoning, this kind of fast and intuitive process (Type 1 or T1) underpins a default response that can only be remediated by the effortful and more demanding Type 2 (T2) process (Evans and Stanovich 2013). Indeed, there is a well-known research tradition in which T2 processes are viewed as being designed to correct or counteract T1 outputs, enabling the reasoner to apply relevant rules of logic and probability that will lead to less harmful, undesirable, irrational, or impulsive courses of actions (Kahneman 2003). However, this research tradition has been focusing on T1 processes as the source of the decision-making error, mostly ignoring the potential contribution of decision-making errors associated with T2.

In this paper, we aimed to uncover judgment errors associated with T2. In particular, we are interested in an overgeneralization bias, whereby the agent continues to deliberatively apply rules of probability in circumstances in which it may no longer be appropriate to do so. We suggest that the misuse of probability rules is more likely to occur when T2 processing is based on a superficial understanding of normative principles. For instance, when one inhibits T1 intuitive responses to make deliberate use of biased statistics. 

Revisiting our opening example, if the problem included an additional premise stating that in the first day of the study the psychologists had randomly chosen, say, two lawyers and two engineers to interview (from the total sample of lawyers and engineers), then the same number of lawyers and engineers would have been interviewed, and therefore the base rates corresponding to the initial group composition (995 lawyers and 5 engineers) are not relevant. Hence, considering those base rates would be a mistake.

In what follows, we begin by briefly reviewing relevant research on dual-process models. Next, we focus on how the overuse of rules (i.e., relying on rules of probability in circumstances where it is inappropriate to do so) may lead to errors and biases (Elqayam and Evans 2011; Fong et al. 1986). We will then describe and test a way to experimentally discriminate between the suitable and unsuitable use of rules, using the conflict-detection paradigm (e.g., De Neys and Pennycook 2019) with a variation of base-rate problems better capable of capturing unwarranted, rule-based thinking.

A major source of experimental evidence to test and contrast different theories of reasoning comes from research using reasoning tasks, where a stereotypical or belief-based heuristic response conflicts with a normatively correct response that requires the use of probabilistic or logical principles. One classic example of these tasks is the base-rate neglect task, where the description of an individual target is inconsistent with the majority of the sample from which the description was drawn (e.g., Kahneman and Tversky 1973; De Neys and Glumicic 2008).

In responding to these tasks, T2 thinking is often seen as being responsible for the application of relevant rules of logic and probability and is thus associated with normatively correct responses, whereas belief biases are attributed to T1 processes and to failures in engaging in T2 thinking (necessary to override the biases; Kahneman 2003; Kahneman and Frederick 2002; Stanovich 1999; Stanovich and West 1998, 2008). 

Other dual-process approaches present an alternative view. They argue that conflict is usually implicitly detected between different T1 outputs. That is, in addition to the heuristic intuitions that lead to erroneous responses in the “cognitive illusions” tradition (e.g., Gilovich et al. 2002), reasoners also have logical intuitions (i.e., implicit knowledge of the logical and probabilistic principles) that are automatically activated when they respond to reasoning tasks. Response bias may still prevail because reasoners might fail to override the stronger intuitive appeal of the heuristic outputs, eventually leading them to behave against their logical intuitions (De Neys 2012; De Neys and Glumicic 2008; Morsanyi and Handley 2012). 

Several authors (Ball et al. 2017; De Neys 2012, 2018; Handley et al. 2011; Pennycook et al. 2015; Thompson et al. 2018) have begun to successfully integrate the conceptual distinction brought by these different dual-process views in a process model of analytic engagement. According to this model, classic reasoning problems are likely to generate one or more competing T1 outputs (De Neys 2012, 2014). If no conflict is detected (either because T1 processes generate just one output or because the difference in activation strength between the competing intuitions is large enough to avoid conflict detection), heuristic responses will be accepted with cursory T2 processing. If more than one T1 outputs are generated and conflict between them is detected, more substantive T2 reasoning will be engaged, which may lead to confirmation of one of the T1 outputs or the replacement of the initial T1 outputs by a T2 normatively correct response. 

Despite the substantial progress accomplished so far, most previous research and theorizing tends to assume that correct responses to reasoning tasks are logical or probabilistic sound responses either cued by logical intuitions or generated by T2 thinking. 

However, as noted by Elqayam and Evans (2011) (see also, Evans and Stanovich 2013; Stanovich 2009; Thompson et al. 2011), the temptation to treat correct responses as being diagnostic of T2 reasoning is a dangerous “ought-is” fallacy (i.e., inferring from normative responses that T2 processing is involved), which has been described as “perhaps the most persistent fallacy in the perception of dual-process theories” (Evans and Stanovich 2013, p. 229). 

The ought-is fallacy is particularly problematic in classic reasoning tasks where there are just two alternative answers: one considered biased, and one considered correct. Choosing the correct answer in such forced-choice paradigms is often interpreted as the T2 suitable use of normative principles. Furthermore, longer response times and lower response confidence are taken as indicators of conflict detection between the two alternative answers, often followed by an increased level of T2 engagement (e.g., De Neys and Pennycook 2019; Pennycook et al. 2015; Thompson et al. 2011). 

However, engagement in T2 processing is not a sufficient condition for the suitable use of normative principles because T2 processes are not immune to error (Evans 2018; Pennycook et al. 2015; Stanovich 2009; Thompson et al. 2011). T2 thinking may simply be normatively incorrect as it happens, for example, when people draw upon the “law of averages” to justify their belief in the gambler’s fallacy (Elqayam and Evans 2011). Furthermore, the overuse of rules may lead to false alarms. That is, relying on these rules in situations where it is inappropriate to do so (Elqayam and Evans 2011; Fong et al. 1986). 

Given the aforementioned one-to-one correspondence in classic reasoning tasks between T1 heuristic-based processes and incorrect responses, and sound logic (either cued by logic intuitions or generated by T2 rule-based processes) and correct responses, it is often difficult to evaluate whether participants who give the rule-based response are actually making an appropriate use of the underlying normative principles. This is because there is no way to discriminate between the suitable use and the misuse of such principles.

The main goal of the research here reported is to evaluate errors associated with T2 thinking. In particular, we are interested in an overgeneralization bias, whereby reasoners apply rules of probability in circumstances where is not appropriate to do so. This may occur if superficial features of a judgment problem cue the use of rules even when the underlying normative principle is not applicable (e.g., references to base rates in base-rate problems may cue its use even when these are non-applicable). 

To experimentally discriminate between the suitable and unsuitable use of rules, we developed a new set of base-rate problems that may give rise to unwarranted, rule-based thinking, henceforth referred to as rule-inadequate versions of standard problems. These are described in the following section. 

Our approach was inspired by previous work that also made use of new (rule-inadequate) versions of standard reasoning problems (Fong et al. 1986; Klauer and Singmann 2013). These versions are typically made by introducing changes to the deep structure of standard problems (e.g., shifting the logical validity of syllogisms or rendering statistical information of a probabilistic problem invalid) while leaving the problems’ surface features largely unchanged. 

Specifically, to test the effects of statistical training in the use of normative principles in reasoning, such as the law of large numbers, Fong et al. (1986) developed standard and so-called false-alarm versions of reasoning problems opposing statistical information to stereotype-based information. In this case, false-alarm answers involved (inadequate) conclusions drawn from large but biased samples. This was crucial to show that even brief sessions of statistical training increased the use of the law of large numbers in standard problems without increasing its use in situations where it would be inappropriate to do so (i.e., rule-inadequate problems).

In a similar vein, Klauer and Singmann (2013) developed “pseudo-syllogisms”, which may be described as rule-inadequate versions of standard syllogism problems. Indeed, pseudo-syllogisms modify the logical status of the original syllogisms while keeping the superficial structure of the problems (formal features and contents) unchanged. The use of “pseudo-syllogisms” allowed Klauer and Singmann to reinterpret previous findings that people can intuitively detect the logicality even of difficult syllogisms (Morsanyi and Handley 2012). We will return to this point in the General Discussion.

### How to Discriminate between the Suitable and Unsuitable Use of Normative Rules in the Base-Rate Neglect Task

To experimentally distinguish between reasoning that makes an appropriate use of rules and reasoning that uses rules in an indiscriminate fashion, we used the conflict-detection paradigm and invited participants to respond not only to standard (conflict and no-conflict) versions of base-rate problems but also to rule-inadequate versions of these problems. The latter present a conflict between stereotypical information and probabilistic information in the form of invalid or irrelevant base rates that do not provide a valid basis for decisions. That is to say, the conflict in rule-inadequate problems is only apparent or spurious.

To illustrate, take the well-known lawyer–engineer problem (Kahneman and Tversky 1972) presented in the beginning. A rule-inadequate version of the same problem would be as follows:

*In a study, several psychologists interviewed a group of people. The group included 5 engineers and 995 lawyers. **In the first day of the study, an equal number of engineers and lawyers were interviewed**, and the psychologists prepared a brief summary of their impression of each interviewee. The following description was drawn randomly from this set of descriptions: Dan is 45. He is conservative, careful, and ambitious. He shows no interest in political issues and spends most of his free time on his many hobbies, which include carpentry, sailing, and mathematical puzzles. Which of the following is more likely?*

* (a) Dan is an engineer*

* (b) Dan is a lawyer*

In this version, there is additional information indicating that the base rates (stemming from the group composition: 5 engineers and 995 lawyers) may not be reliable in the current context. For those such as Dan, who were tested on the first day, the prior probability of being an engineer or a lawyer is the same (i.e., 50/50). If participants go ahead and still use the initial base rates to respond, this would be an inappropriate use of the base-rate information.

Sound statistical thinking should discriminate between standard and rule-inadequate versions of base-rate problems, taking into consideration the 5/995 group composition in the former but discarding them in the latter (leaving Dan’s description as the only source of diagnostic information). 

In contrast, an indiscriminate reliance on the base rates of the group composition (5/995) is expected to lead to responding according to them both in the standard and rule-inadequate versions of the problem. 

Importantly, the conflict-detection paradigm further allowed us to look into the participants’ metacognition. More specifically, whether participants show some sensitivity to the opposing information in standard conflict problems (e.g., De Neys and Glumicic 2008). 

If participants do not take logical principles into account (e.g., neglecting base-rate information), then conflict should be irrelevant and have no impact on reasoning. However, previous findings indicate that biased reasoners often do show conflict sensitivity by displaying increased response doubt. This is reflected in longer response latencies and lower confidence in their incorrect answers to conflict problems compared to correct answers to no-conflict problems (De Neys 2012; De Neys and Glumicic 2008; Pennycook et al. 2015). 

We expect to observe the same kind of conflict effects with rule-inadequate versions of base-rate problems as have been observed with standard base-rate problems. That is, we predict that the irrelevant base rates in rule-inadequate problems are going to trigger (inaccurate) logical intuitions that oppose other T1 intuitions (based on the similarity between the target’s description and a professional/group stereotype). This may lead to the detection of a spurious conflict between invalid base rates and stereotype-based information that will be reflected by longer responses latencies (Experiments 1 to 4) and lower response confidence (Experiment 4).

Finally, individual differences in reflective thinking ability have been shown to be associated with judgment biases and errors (e.g., Toplak et al. 2011). We thus used the cognitive reflection test (CRT; Frederick 2005) to assess participants’ susceptibility to the overgeneralization bias. We expect higher reflective ability to be associated with less overgeneralization bias.

## 2. Experiment 1

Experiment 1 is a first attempt at examining the extent to which base-rate responses to standard problems stem from adequate statistical thinking or from an indiscriminate use of base rates (i.e., using the base rates both when they are valid and invalid). In order to obtain measures of processing time, participants responded to a “moving window” paradigm (De Neys and Glumicic 2008; Just et al. 1982), in which there were three kinds of problems: standard base-rate problems—conflict and no-conflict versions—and conflict versions of rule-inadequate problems, which present a spurious conflict between base rates and stereotype-based information. In this paradigm, the base-rate information and the stereotypical description are presented separately. Participants were first presented the base-rate information on a computer screen, which was then substituted by the stereotypical description and the question. Before giving their answer, participants had the option to visualize the base-rate information again by pressing and holding down a specific computer key. 

De Neys and Glumicic (2008) showed that not only normatively correct responses but also incorrect responses to problems that presented a conflict between the description and the previously presented base rates took longer (and were associated with more revisits of the initial base-rate information) than responses to no-conflict problems (where base rates were congruent with the stereotype description), thus providing evidence consistent with conflict detection even for participants who gave the heuristic response.

Our main predictions are as follows. An indication of the indiscriminate use of base-rate information might be found in the form of a positive correlation between base-rate responses for standard and rule-inadequate problem versions. This would show that the more participants give base-rate responses to standard problems, the more they overgeneralize the use of base rates to cases where the base rates are invalid (i.e., rule-inadequate problems)^2^. Furthermore, detection of (a spurious) conflict between the stereotype-based information and irrelevant base rates (followed by unwarranted use of these base rates) should be apparent if base-rate responses to rule-inadequate problems are also coupled with longer response times and a higher frequency of base rate reviews (when compared to no-conflict problems). In addition, participants’ responses to standard versions of base-rate problems are expected to replicate De Neys and Glumicic’s (2008) results.

### 2.1. Method

#### 2.1.1. Participants

Eighty-six psychology undergraduate students (70 female; *M*_age_ = 21.2, *SD* = 6.02) participated in the study in exchange for course credits^3^. Sensitivity analysis with this sample size (at α = .05, and power = .80) showed that the experimental design could reliably detect medium or larger effect sizes (*f ≥* .34).

#### 2.1.2. Material

The material included 18 problems (translated and adapted to Portuguese from De Neys and Glumicic 2008). Three were presented during practice trials, while the remaining fifteen were used in the experimental task. The problems followed the common design of base-rate problems, with a description of the base rates for two groups of people and a description of characteristics of a subject selected randomly from those groups. The description was either stereotypical of the larger group (no-conflict problems) or of the smaller group (conflict problems). The original problems were changed to include information that rendered the described base rates irrelevant (rule-inadequate problems) or kept these base rates relevant (standard problems). The following is an example of one of the problems, with the text that renders the base rates irrelevant in italics:

In a study 1000 people were tested. Among the participants there were 996 women and 4 men. *In the first week of the study, as many men as women were interviewed. Jo was one of the people interviewed in the first week.*

Jo is 23 years old and is finishing a degree in engineering. On Friday nights, Jo likes to go out cruising with friends while listening to loud music and drinking beer.

 What is most likely?

 a. Jo is a man

 b. Jo is a woman

In this case, the tested subsample comprises the same number of men and women, so the *relevant* base rates indicate a 50/50 chance (rather than the initial 996/4) for the described person to be a woman or a man. 

For standard problems, the phrase in italics reads, “*In the first week of the study, all men and women were interviewed*.” As such, the relevant base rate is still 996/4, making “b” (“Jo is a woman”) the most likely correct response (examples for all variations of problems in Experiment 1 are displayed in Appendix B, Table A3).

The fifteen problems were separated into three sets of five problems each. In each set, the problems were all of one type: standard conflict problems, standard no-conflict problems, or rule-inadequate problems. 

Problem content was counterbalanced across problem types and not repeated for an individual participant. Conflict and no-conflict versions of standard problems were exactly the same except for the base rates, which were inverted. Rule-inadequate versions were the same as conflict versions, except that the original base rates were rendered irrelevant with the addition of the subsample information (each participant responded to only one of the three versions of each specific problem). All problems presented extreme base rates with three variations between them (997/3, 996/4, or 995/5), which were assigned evenly between the fifteen problems.

#### 2.1.3. Procedure

Participants performed the experiment on a computer, where they received instructions to read the texts and to solve the problems at their own pace. Participants were presented 3 practice trials. 

Participants were informed that, in each trial, they would see the first part of the problem (containing the base-rate information). After reading the text and whenever they felt ready to move on, they should press the space key on the keyboard to replace the base-rate information with the second part of the problem (containing the stereotypical description of the selected individual and the response options). During the display of this second part, participants could again have access to and review the first part with the crucial base-rate information by pressing the space key. As long as they held down the space key, the first part remained visible. Once the space key was released, the information disappeared again. The second part of the text with the description always remained visible after the initial presentation. 

The main dependent variables included the proportion of base-rate responses for each type of problem: the base-rate reading time (when this information was first presented without the stereotype information) and the decision-making time (i.e., the time elapsed between the display of stereotype information and response). The mean number of problems where base-rate information was reviewed was also analyzed for each type of problem.

### 2.2. Results 

In this and the remaining experiments, we begin by describing and analyzing the proportion of base-rate responses across the experimental conditions (see Appendix B, Table A1), as well as the correlation of base-rate responses between standard and rule-inadequate versions of the conflict problems (see Table 1). Evidence for the overgeneralization bias is provided by positive and significant correlations between the two. 

Furthermore, to test for conflict detection, we then analyze response times (Experiment 1 to 4; see Appendix B, Table A2), mean base-rate reviewing (only Experiment 1), and response confidence (only Experiment 4) separately for standard and rule-inadequate problems. If increased response doubt occurs, not only for standard, but also for rule-inadequate problems, this will be an indication of sensitivity to a spurious conflict between response outputs based on stereotypical descriptions and opposing (but irrelevant) base rates (see Table 2 for a summary of the conflict detection results).

#### 2.2.1. Base-Rate Responses

**Figure 1:**
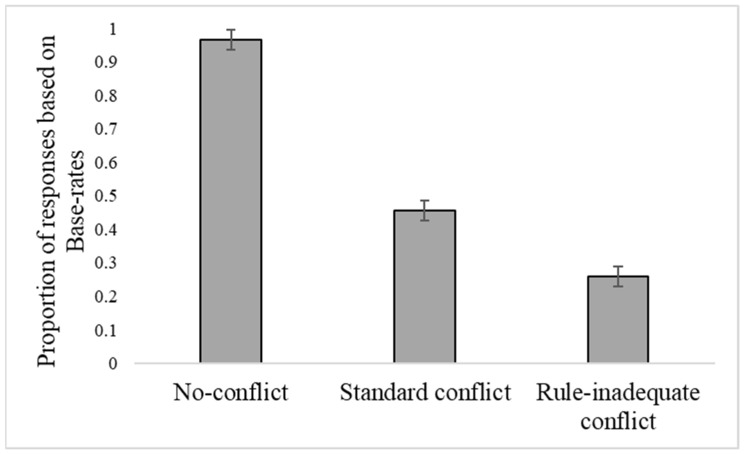
Proportion of correct responses to no-conflict problems^4^ and base-rate responses to the standard and rule-inadequate versions of conflict problems (Experiment 1).

Figure 1 presents the mean proportion of responses according to the base rates in no-conflict problems, and standard and rule-inadequate versions of conflict problems. 

Differences in responses across problems were significant (*F*(2, 170) = 169.36, *p* < .001, η_p_^2^ = .67). Almost all responses were according to the base rates (and stereotype-based information) in no-conflict problems (*M* = .97; *SE* = .03). About half of the responses to conflict problems (*M* = .46; *SE* = .03) and one-fourth of the responses to rule-inadequate problems (*M* = .26; *SE* = .03) were according to base rates. This greater reliance on valid (compared to invalid) base rates may indicate some sensitivity to the quality of the base-rate information at the aggregate level of analysis.

However, and as predicted, the correlation between standard and rule-inadequate problems was positive (*r* = .46, *p* < .001), such that the more participants decided according to base rates in standard problems, the more they did so in rule-inadequate problems. This suggests an overgeneralization bias in the use of base-rate information.

#### 2.2.2. Base-Rate Reading Time

The initial base-rate reading time (i.e., the time people initially spent reading the first part of the problem before the description was presented) did not vary for the three types of decisions (*F* < 1), which indicates that the initial presentation of the base-rate information was not processed differently across problem versions. 

#### 2.2.3. Response Time for Standard Problems

A repeated-measures ANOVA on response times, for the correct responses to no-conflict problems, and stereotype-based responses and base-rate responses to conflict problems as a within-participants factor, revealed a significant effect (*F*(2, 86) = 6.23, *p* = .002, η_p_^2^ = .12). As expected, base-rate responses for conflict problems (*M* = 12,386.22, *SE* = 839.41) were slower than base-rate responses to no-conflict problems (*M* = 9615.04, *SE* = 839.41; *F*(1, 43) = 10.96, *p* = .001, η_p_^2^ = .20). 

Stereotype responses for conflict problems (*M* = 12,052.92, *SE* = 839.41) were also slower than stereotype responses to no-conflict problems (*F*(1, 43) = 10.46, *p* = .002, η_p_^2^ = .19), providing evidence consistent with conflict detection for participants who gave the heuristic response (see Appendix B, Table A2).

#### 2.2.4. Response Time for Rule-Inadequate Problems

A repeated measure ANOVA on response times, with correct responses to no-conflict problems, and stereotype responses and base-rate responses to rule-inadequate problems as a within-participants factor, did not yield a significant effect (*F*(2, 66) = 1.91, *p* = .157, η_p_^2^ = .05). Likewise, the tendency for longer times in base-rate responses to conflict problems (*M* = 11,995.65, *SE* = 857.88) than to correct responses to no-conflict problems (*M* = 10,117.63, *SE* = 857.88) did not reach conventional levels of statistical significance (*F*(1, 33) = 3.89, *p* = .057, η_p_^2^ = .10). Finally, the difference between stereotype-based responses to conflict problems (*M* = 10,987.56, *SE* = 857.88) and no-conflict problems was not significant (*F*(1, 33) = 1.84, *p* = .184, η_p_^2^ = .05) (see Appendix B, Table A2).

#### 2.2.5. Base-Rate Reviewing for Standard Problems

A repeated-measures ANOVA, with an average number of problems reviewed for correct responses to no-conflict problems and base-rate and stereotype-based responses to standard conflict problems, did not reach significance (*F*(2, 90) = 2.59, *p* = .080, η_p_^2^ = .05). The reviewing tendency of base-rate information was higher for base-rate responses to conflict problems (*M* = .43, *SE* = .06) than for correct responses to no-conflict problems (*M* = .33, *SE* = .49) but was also non-significant (*F*(1, 45) = 3.35, *p* = .074, η_p_^2^ = .06). There was no difference in reviewing between stereotype-based responses to conflict problems (*M* = .31, *SE* = .05) compared to correct responses to no-conflict problems (*F* < 1).

#### 2.2.6. Base-Rate Reviewing Rule-Inadequate Problems

An equivalent ANOVA, performed with rule-inadequate problems, produced a significant effect (*F*(2, 68) = 5.41, *p* = .006, η_p_^2^ = .13). Base-rate responses to conflict problems (*M* = .45, *SE* = .08) had more reviews than correct responses to no-conflict problems (*M* = .36, *SE* = .06; *F*(1, 34) = 4.38, *p* = .043, η_p_^2^ = .11). 

Reviews of rule-inadequate problems with stereotype responses (*M* = .26, *SE* = .04) did not significantly differ from reviews of no-conflict problems (*F*(1, 34) = 2.32, *p* = .136, η_p_^2^ = .06). 

### 2.3. Discussion

A larger number of participants used base rates when they were a valid source of information (standard problems) than when they were not (rule-inadequate problems), suggesting some discrimination between relevant and irrelevant base-rate information. Nevertheless, the mean frequency of responses in the latter category (26%) is non-negligible. Importantly, the positive correlation between base-rate responses to standard and rule-inadequate problems is congruent with an overgeneralization bias in the use of base-rate information. Indeed, those participants who relied more on base rates to respond to standard problems also relied more on base rates to respond to rule-inadequate problems.

In addition, response latencies (but not review frequency) provided some indication of conflict detection and possible engagement of T2 reasoning for responses to standard problems, whereas review frequency (but not response latencies) provided some indication of conflict detection and possible engagement of T2 for responses to rule-inadequate problems (see Table 2). 

Taken together, these initial findings provide preliminary indications of an overgeneralization bias in the use of base rates and may be seen as questioning the extent to which base-rate responses to the base-rate problems rely on the proper use of the statistical information (as is often assumed).

After having established the basic phenomenon, we sought to increase the number of observations that we could make per person, as well as to potentially reduce the large variability in response time that arose because of the lengthy text passages that we used. Indeed, response times in classic base-rate problems tend to be quite noisy, with the mean RT range varying between 3.4 s and 21.80 s. This variability may have made it difficult to detect the subtle conflict effects.

Furthermore, standard no-conflict problems were used as a baseline for standard conflict and rule-inadequate problems. A better baseline for the latter would be no-conflict versions of rule-inadequate problems, which were included in the following experiments. 

## 3. Experiment 2

Experiments 2 used a rapid-response base-rate paradigm (Pennycook et al. 2015). In this paradigm, base rates and stereotype-based information are presented in a simplified manner in a fast-paced sequence of computer screens. 

On the first screen, the two groups involved in the problem were presented (e.g., “politicians and nannies”); the second screen presented an attribute (e.g., “kind”) pertaining to the target subject who was randomly drawn from the total sample of the two groups (e.g., “politicians and nannies”); the third screen presented the groups’ composition (e.g., “995 politicians and 5 nannies”); and the fourth screen presented the question and response options (e.g., “is the target person more likely to be a politician or a nanny?”). 

This paradigm has been shown to be sensitive to conflict detection and subtle increases in T2 thinking (Pennycook et al. 2015) and allows us to substantially increase the number of trials (base-rate problems) per participant. 

**Figure A1:**
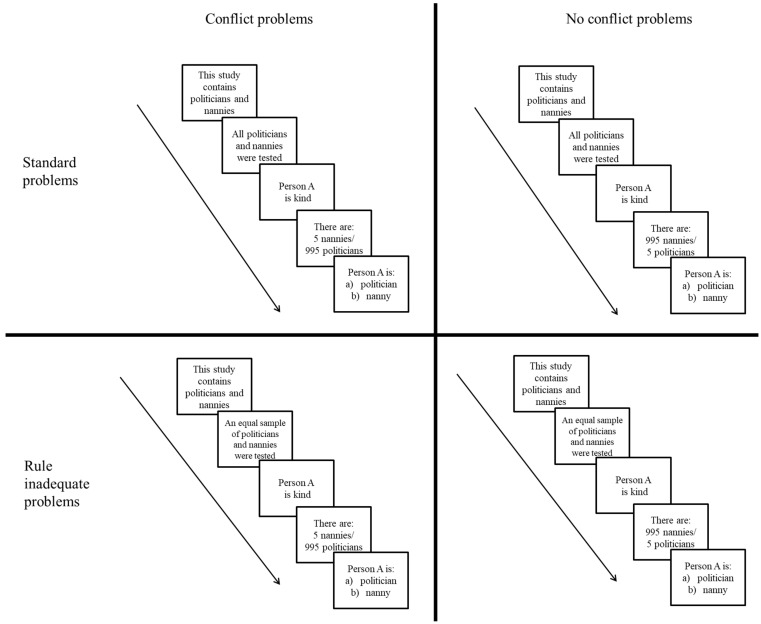
Examples for all variations of problems used in Experiment 2.

Furthermore, whereas Experiment 1 used three types of problems (i.e., conflict and no-conflict versions of standard problems and conflict versions of rule-inadequate problems), this experiment and the following (i.e., Experiments 3 and 4) also included no-conflict versions of rule-inadequate problems. No-conflict rule-inadequate problems have the same structure as the conflict rule-inadequate problems (i.e., inclusion of additional information that makes the initial base rates irrelevant). However, the initial base rates are aligned with the stereotype-based information. For an outline of these different kinds of problems (standard and rule-inadequate versions of conflict and no-conflict problems), see Table 3. For examples of the problems, see Appendix B, Figure A1.

As in Experiment 1, we predict a positive correlation between base-rate responses for standard and rule-inadequate problem versions (i.e., the more participants give base-rate responses to standard problems, the more they overgeneralize the use of base rates to cases where the base rates are invalid). An unwarranted reliance on irrelevant base rates should also be apparent if base-rate responses to rule-inadequate problems are coupled with longer response times (when compared to no-conflict problems), suggesting the detection of a spurious conflict.

Finally, in Experiments 2 to 4, the CRT was included as a measure of participants’ analytical skills. Performance in the CRT should predict a more appropriate use of base-rate information. 

### 3.1. Method

#### 3.1.1. Participants

One hundred-sixteen students from the University of Heidelberg (89 female) performed the experiment for course credits. Sensitivity analysis with this sample size (at α = .05, and power = .80) showed that the experimental design could reliably detect medium or larger effect sizes (*f ≥* .31).

#### 3.1.2. Material

Sixty-six pairs of social groups, with opposite stereotype personality traits associated with each group pair, were obtained through pre-testing. In the pre-test, an independent sample of 40 students was presented with a sample of groups (including professions and sociodemographic categories) and a sample of personality traits^5^. Participants were asked to indicate the two traits most stereotypical of each group. The groups were subsequently paired, and two personality traits were selected so that one trait was frequently associated with one group and rarely (if ever) associated with the other, and vice versa for the other selected trait. Twenty-two pairs were presented in the rule-inadequate version and forty-four pairs were presented in the standard version^6^. In each version, half were conflict trials, and the other half were no-conflict trials. Content was counterbalanced across problem type by creating two lists of trials that varied only in the way the base rates were presented, so that pairs of a conflict type of problem in one list were of a no-conflict type of problem in the other, and vice versa. Participants were randomly presented with one of the two lists of problems. 

#### 3.1.3. Procedure

The procedure was based on Pennycook et al.’s (2015) rapid-response base-rate task, with minor adjustments to accommodate the inclusion of rule-inadequate versions of both no-conflict and conflict problems. Specifically, the base rates were qualified by further information referring to how many people from each of the two groups (i.e., initial base rates) were actually tested: the whole sample (in the case of standard trials) or a subsample composed of an equal number of individuals from each of the two groups (in the case of rule-inadequate trials). Participants were also informed that the individual described in each trial was randomly selected from the tested sample.

Participants began by reading the following instructions taken from Pennycook et al. (2015; text in italics corresponds to additional sentences introduced to accommodate the use of rule-inadequate problems):

In a big research project, a large number of studies were carried out where short personality descriptions of the participants were made. In every study, there were participants from two population groups (e.g., carpenters and policemen). In each study, one participant was drawn at random from *a sample of tested participants*. In each trial, you will be presented with a fixation dot where you should look at. After a few seconds, this dot will be replaced by a personality trait for the randomly chosen participant and finally by some information about the composition of the population groups *and how many participants from each group were tested in the study in question*. After that, you will be asked to indicate to which population group the participant most likely belongs. Please answer the problems as quickly and accurately as possible. Once you have made up your mind, you must enter your answer (‘a’ or ‘b’) immediately and then the next problem will be presented.

**Figure 2:**
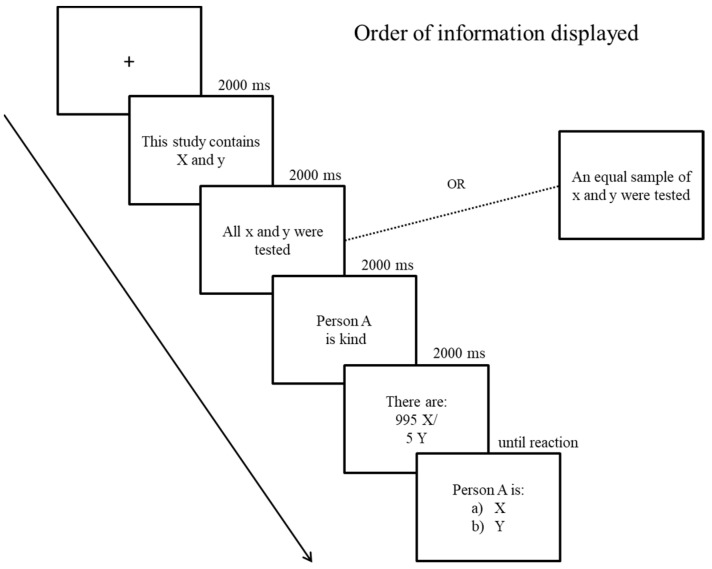
Procedure for rapid-response base-rate task (Experiment 2).

Following Pennycook et al. (2015), each trial began with the presentation of a fixation point (500 ms), followed by (a) the information concerning the groups involved in the trial (2000 ms), (b) the number of individuals actually tested (2000 ms), (c) the trait describing one of the individuals tested (2000 ms), and (d) the groups’ base rates (2000 ms). Participants were then asked to which group the individual most likely belonged (see Figure 2).

Participants began by responding to three practice trials, after which they responded to the block of sixty-six trials (each trial corresponding to a different group pair). After the experimental task, participants responded to the CRT (see Appendix A).

Dependent measures included base-rate responses for each type of problem, as well as response time (RT) of correct responses to no-conflict problems and of stereotype-based and base-rate responses to conflict problems.

### 3.2. Results

#### 3.2.1. Base-Rate Responses

**Figure 3:**
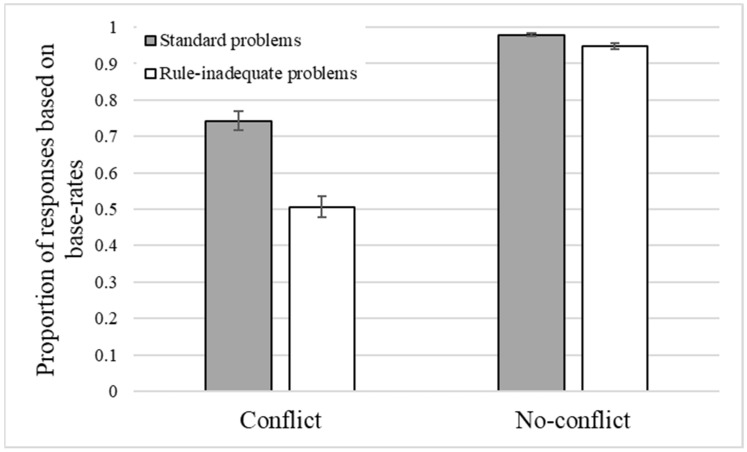
Proportion of correct responses to no-conflict problems and base-rate responses to the standard and rule-inadequate versions of conflict problems in Experiment 2.

Three participants with accuracies below .80 in the no-conflict standard trials were removed from the analysis (see Pennycook et al. 2015). The mean proportion of base-rate responses for conflict and no-conflict problems for standard and rule-inadequate problems is presented in Figure 3.

A 2X2 ANOVA, with trial version (standard and rule-inadequate) and trial type (conflict and no-conflict) as within-participant factors, and responses based on base rates as the dependent variable, showed a significant main effect of version (*F*(1, 112) = 85.65, *p* < .001, η_p_^2^ = .43), with a higher frequency of base-rate responses for standard problems (*M* = .86, *SE* = .02) than for rule-inadequate ones (*M* = .73, *SE* = .02). A significant main effect of trial type was also observed (*F*(1, 112) = 211.78, *p* < .001, η_p_^2^ = .65), with a higher proportion of base-rate responses for no-conflict problems (*M* = .96, *SE* = .02) than conflict ones (*M* = .62, *SE* = .02). Additionally, there was a significant interaction between the factors (*F*(1, 112) = 50.75, *p* < .001, η_p_^2^ = .31), indicating that the difference between standard and rule-inadequate problems was larger for conflict problems (*M* = .74, *SE* = .03 for standard problems, and *M* = .51, *SE* = .03 for rule-inadequate problems) than for no-conflict problems (*M* = .98, *SE* < .01 for standard problems, and *M* = .95, *SE* = .01 for rule-inadequate problems (see Appendix B, Table A1).

Overall, the greater reliance on valid (compared to invalid) base rates indicates some sensitivity to the quality of the base-rate information at the aggregate level of analysis.

However, there was a large positive correlation between standard and rule-inadequate trial versions (*r* = 50; see Table 1), indicating that the more participants decided according to base rates in standard trials, the more they did so in rule-inadequate trials, suggesting an overgeneralization bias in the use of base-rate information.

Furthermore, in order to verify whether more reflective participants were better able to discriminate between trials where base rates were a relevant source of information (standard trials) and trials where they were not (rule-inadequate trials), participants were divided in two subgroups based on their CRT performance. 

The low-reflective reasoners included participants who gave one or more incorrect responses to the CRT (*N* = 70) and the high-reflective reasoners included participants who responded correctly to all three CRT problems (*N* = 43). 

The rationale for this partition was that those participants who respond to all problems correctly were inhibiting and replacing the highly appealing intuitive responses with the analytical correct responses in a consistent way (whereas the remaining participants did it inconsistently or not at all across the three CRT problems). As such, these are the participants with better chances to discriminate between standard and rule-inadequate base-rate problems (i.e., whether or not to inhibit the stereotype-based response). 

For low-reflective reasoners, we found a strong positive correlation between base-rate responses to conflict standard and rule-inadequate trials (*r* = .70), whereas for high-reflective reasoners there was no correlation (*r* = .05). High-reflective participants showed a higher frequency of base-rate responses for standard conflict problems compared to rule-inadequate conflict problems (difference of .30) than low-reflective participants (.19). See Table 4 for details.

#### 3.2.2. Response Times

Response times (converted to log_10_) were analyzed separately for standard and rule-inadequate trials (see Appendix B, Table A2 for means in ms). 

*Standard trials*. A repeated-measures one-way ANOVA, with base-rate responses to no-conflict trials, and base-rate and stereotype-based responses to conflict trials, yielded a significant main effect (*F*(2, 166) = 41.94, *p* < .001, η_p_^2^ = .34). Planned comparisons showed that the RT for no-conflict trials (*M* = 2.96, *SE* = .02) was faster than base-rate responses to standard conflict trials (*M* = 3.08, *SE* = .03; *F*(1, 83) = 40.18, *p* < .001, η_p_^2^ = .33) and faster than stereotype-based responses to standard conflict trials (*M* = 3.17, *SE* = .03; *F*(1, 83) = 86.40, *p* < .001, η_p_^2^ = .51). 

This pattern of results replicates previous findings by Pennycook et al. (2015), suggesting that responding according to base rates involved the engagement in time-consuming, deliberate reasoning. Furthermore, stereotype-based responses to conflict trials were also slower than to no-conflict trials, providing evidence consistent with conflict detection (between stereotype and base-rate information) for participants who ended up giving the heuristic response.

*Rule-inadequate trials*. A repeated-measures ANOVA, with base-rate responses for no-conflict trials, and base-rate and stereotype-based responses for conflict trials, yielded a significant main effect (*F*(2, 186) = 13.54, *p* < .001, η_p_^2^ = .13). Planned comparisons showed that responses to no-conflict trials (*M* = 3.02, *SE* = .03) were faster than base-rate responses to rule-inadequate conflict trials (*M* = 3.16, *SE* = .04; *F*(1, 93) = 24.78, *p* < .001, η_p_^2^ = .21) and faster than stereotype-based responses to rule-inadequate conflict trials (*M* = 3.14, *SE* = .03; *F*(1, 93) = 20.01, *p* < .001, η_p_^2^ = .18). 

In other words, the response-time pattern that was obtained for the standard trials was also obtained for rule-inadequate trials. This suggests that responding according to invalid base rates also involved conflict detection and possible engagement in T2 thinking. Furthermore, there is also indication that participants detected a conflict between traits and opposite (but invalid) base-rate information, even when they opted for the heuristic response.

### 3.3. Discussion

The number of participants who responded according to the base rates when they were a valid source of information (standard problems) was larger than when the base rates were made irrelevant (rule-inadequate problems). This suggests some discrimination between relevant and irrelevant base-rate information. However, the mean frequency of responses in the latter category (51%) is considerable. In addition, the positive correlation between base-rate responses to standard and rule-inadequate problems is congruent with an overgeneralization bias in the use of base-rate information.

The positive correlation in the tendency to respond according to base rates observed between standard and rule-inadequate conflict problems was, however, weaker for highly reflective participants compared to less reflective participants. This could suggest individual differences in the expected direction concerning the relation between rationality (as measured by the CRT) and the discriminate use of statistical information.

Response-time analysis of standard problems provided evidence of conflict detection, both when participants gave the stereotype-based response and when they responded according to the base rates. The same results pattern emerged for rule-inadequate problems, where there is no real conflict between statistical information and stereotype-based information. Taken together, these results suggest that participants failed to discriminate between real and merely apparent (or spurious) conflict and misused statistical information to draw conclusions based on irrelevant base rates. 

## 4. Experiment 3

Experiment 2 presented the group compositions last, as in the original studies with this rapid-response paradigm (Pennycook et al. 2015, Experiments 1 to 3). 

However, previous research has shown that presenting a piece of information last, just before judgment, increases the likelihood of its use (Krosnick et al. 1990; Pennycook et al. 2015, Experiment 4). It is thus possible that including the subsample (vs. the group composition) information as the last piece of information prior to judgment would increase the adequate use of base rates. In Experiments 3 and 4, the information about the subsample actually tested and the group compositions was presented in the last two screens (before the screen with the question) and its order of presentation was manipulated. 

### 4.1. Method

#### 4.1.1. Participants

Eighty-six students from the University of Heidelberg (71 female) completed the experiment in exchange for course credit. Sensitivity analysis with this sample size (at α = .05, and power = .80) showed that the experimental design could reliably detect medium or larger effect sizes (*f ≥* .36).

#### 4.1.2. Material

Forty-four pairs of social groups were selected from the material used in Experiment 2, with opposite stereotypical traits associated with each group pair. Half of the pairs were assigned to standard problems and the other half to rule-inadequate problems. Both kinds of problems had conflict and no-conflict versions (obtained by changing the personality trait associated with the trial).

#### 4.1.3. Procedure

**Figure 4:**
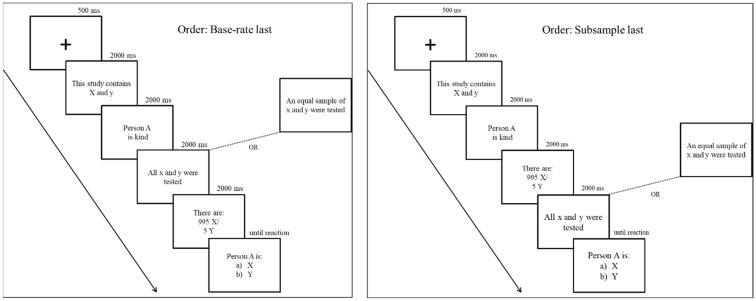
Procedure for rapid-response base-rate task (Experiment 3).

The procedure was the same of Experiment 2. However, the order of presentation of the base rates for each trial and the information referring to how many people from each of the two groups were actually tested—the whole sample (in the case of standard base-rate problems) or a subsample composed of an equal number of individuals from each of the two groups (in the case of rule-inadequate problems)—was manipulated. Participants in the present study either saw the base rates followed by the subsample of individuals who were actually tested or the other way around (subsample information followed by the base rates from where the subsample was taken) (see Figure 4).

Participants began by responding to three practice trials, after which they responded to 2 blocks of 44 trials (each trial corresponding to a different group pair). Between blocks, the pairs were the same, but the trait presented for each pair varied so that a conflict problem in one block would be a no-conflict problem in the other block and vice versa. After the experimental task, participants responded to the CRT.

### 4.2. Results

#### 4.2.1. Base-Rate Responses

**Figure 5:**
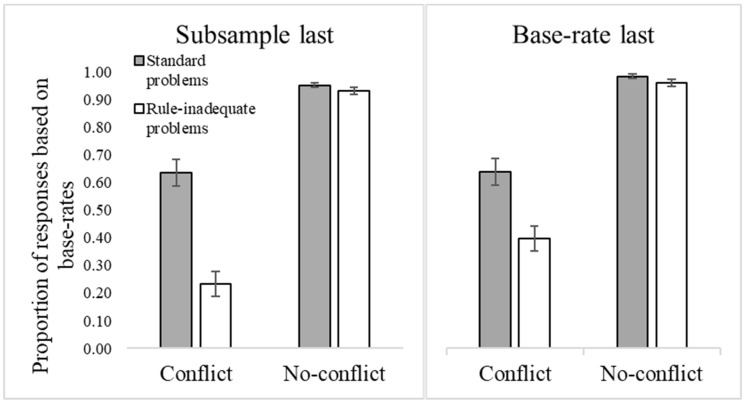
Proportion of correct responses to no-conflict problems and base-rate responses to the standard and rule-inadequate versions of conflict problems in both orders of information presentation (Experiment 3).

One participant with an accuracy below .80 in the no-conflict standard problems was removed from the analysis. The mean proportion of base-rate responses for conflict and no-conflict problems for standard and rule-inadequate problems is presented in Figure 5.

A 2X2X2 ANOVA, with problem version (standard and rule-inadequate problems) and trial type (conflict and no-conflict) as within-participant factors, with order (subsample last; base rate last) as the between-participant factor, and with the mean proportion of base-rate responses as the dependent variable, showed a main effect of trial type, with a higher proportion of base-rate responses for no-conflict problems (*M* = .96, *SE* = .01) than conflict ones (*M* = .48, *SE* = .03; *F*(1, 83) = 261.09, *p* < .001, η_p_^2^ = .76) and a main effect of version (*F*(1, 83) = 112.22, *p* < .001, η_p_^2^ = .58), with a higher proportion of base-rate responses to standard problems (*M* = .80, *SE* = .02) than to rule-inadequate problems (*M* = .63, *SE* = .02). There was a version x order interaction (*F*(1, 83) = 5.86, *p* = .018, η_p_^2^ = .07). While in standard problems, the frequency of base-rate responses did not differ significantly between different orders of information (*F* < 1) for rule-inadequate problems, the frequency of base-rate responses was lower when the subsample information was presented last (*M* = .44, *SE* = .04) compared to when base rates were presented last (*M* = .52, *SE* = .04; *F*(1, 83) = 8.68, *p* = .004, η_p_^2^ = .09). There was also a version x type interaction (*F*(1, 83) = 84.82, *p* < .001, η_p_^2^ = .51). The difference in base-rate responses between standard (*M* = .64, *SE* = .03) and rule-inadequate problems (*M* = .32, *SE* = .03) was higher for conflict problems (*F*(1, 83) = 104.84, *p* < .001, η_p_^2^ = .56) than for no-conflict problems (*M* = .97, *SE* = .01 for standard problems, and *M* = .95, *SE* = .01 for rule-inadequate problems; *F*(1, 83) = 7.70, *p* = .007, η_p_^2^ = .08). Finally, there was a 3-way interaction between version, type, and order (*F*(1, 83) = 6.47, *p* = .013, η_p_^2^ = .07). Planned comparisons showed that the proportion of base-rate responses in conflict problems was lower when the subsample information was presented last for rule-inadequate problems (*F*(1, 83) = 6.62, *p* = .012, η_p_^2^ = .07), but not for standard problems (*F* < 1). Regarding no-conflict problems, the difference in base-rate responses was also lower when the subsample information was presented last for standard problems (*F*(1, 83) = 6.13, *p* = .015, η_p_^2^ = .07), but not for rule-inadequate problems (*F*(1, 83) = 2.55, *p* = .114, η_p_^2^ = .03) (see Appendix B, Table A1).

In sum, the analysis of base-rate responses replicated that of Experiment 2. Furthermore, when the subsample of participants actually tested was the last information presented, it reduced participants’ reliance on irrelevant base rates (in rule-inadequate problems). 

Regardless, the proportion of base-rate responses to standard and rule-inadequate conflict problems were positively and strongly correlated (*r* = .53, *p* < .001), indicating an overgeneralization bias (see Table 1). The same positive correlation was observed for participants in the subsample-last condition (*r* = .40, *p* = .008) and in the base-rate last condition (*r* = .65, *p* < .001).

In order to verify whether more reflective participants were better able to discriminate between trials where base rates were a relevant source of information (standard problems) and trials where they were not (rule-inadequate problems), participants were divided in two subgroups based on their CRT performance. The low-reflective thinking group included participants who gave one or more incorrect responses to the CRT (*N* = 63) and the high-reflective thinking group included participants who responded correctly to all three CRT problems (*N* = 23). 

For low-reflective participants, there was a positive and large correlation (*r* = .65, *p* < .001) in the proportion of base-rate responses for standard and rule-inadequate conflict problems, whereas for high-reflective participants, this correlation was smaller and not statistically significant (*r* = .32, *p* = .138) (see Table 1). Hence, high-reflective thinking (as measured by the CRT) seems to discriminate slightly better between trials where base rates should (standard problems) and should not be used (rule-inadequate problems). Furthermore, when comparing base-rate responses between standard and rule-inadequate problems, high-reflective participants showed a higher frequency of base-rate responses for standard conflict problems compared to rule-inadequate conflict problems (difference of .51) than low-reflective participants (.26). See Table 4 for details.

#### 4.2.2. Response Times

Response times (converted to log_10_) were analyzed separately for each kind of problem (see Appendix B, Table A2 for means in ms). 

*Standard Problems*. A 3X2 ANOVA, with response (correct responses to no-conflict trials, base-rate or stereotype-based responses to conflict trials) as within-participants factor and order (subsamples last; base rates last) as between-participants factor, yielded just a main effect of response (*F*(2, 136) = 24.54, *p* < .001, η_p_^2^ = .26). Planned comparisons showed that responses to no-conflict trials (*M* = 2.974, *SE* = .03) were faster than base-rate responses to conflict trials (*M* = 3.06, *SE* = .03; *F*(1, 68) = 12.62, *p* < .001, η_p_^2^ = .16) and faster than stereotype-based responses to conflict trials (*M* = 3.19, *SE* = .04; *F*(1, 68) = 38.70, *p* < .001, η_p_^2^ = .36). 

*Rule-inadequate Problems*. A 3X2 ANOVA, with response (correct responses to no-conflict trials, base-rate or stereotype-based responses to conflict trials) as within-participants factor and order (subsamples last; base rates last) as between-participants factor, yielded just a main effect of response (*F*(2, 140) = 13.19, *p* < .001, η_p_^2^ = .16). Planned comparisons showed that responses to no-conflict trials (*M* = 2.99, *SE* = .03) had shorter RTs than base-rate responses to conflict trials (*M* = 3.14, *SE* = .04; *F*(1, 70) = 25.74, *p* < .001, η_p_^2^ = .27) and stereotype-based responses to conflict trials (*M* = 3.07, *SE* = .03; *F*(1, 70) = 12.30, *p* < .001, η_p_^2^ = .15). 

### 4.3. Discussion

In Experiment 3, we found differences in the proportion of base-rate responses as a function of the presentation order of the base rates. Specifically, presenting the crucial information about the number of individuals actually tested after the base-rate information and just before participants were prompted to give their response increased the likelihood of its use and thus helped reduced the reliance on irrelevant base rates. In contrast, there were no changes in the proportion of base-rate responses as a function of order of presentation for standard problems. This is also expected since, in these problems, the last two pieces of information converge in establishing the initial group composition as the problems’ relevant base rates (it makes no difference to say “all were tested/from a group of X and Y” vs. “from a group of X and Y/all were tested).

Still, the positive correlation observed in the previous studies between the proportion of base-rate responses to standard and rule-inadequate trials was replicated for participants in the different information orders, which is congruent with an overgeneralization bias. In addition, highly reflective participants showed this tendency to a lesser degree, which suggests individual differences in the expected direction concerning the relation between rationality (as measured by the CRT) and the discriminate use of statistical information. 

As in Experiment 2, response-time analysis of standard trials provided evidence of conflict detection, both when participants gave the stereotype-based responses and when they responded according to base rates. Interestingly, the same pattern of results emerged for rule-inadequate problems. Participants detected a conflict between stereotype-based and base-rate information, even when base rates were made irrelevant to respond to the problem. In other words, conflict detection does not seem to discriminate between valid and irrelevant statistical information.

## 5. Experiment 4

Experiment 4 was designed to replicate and extend the results of Experiments 2 and 3, with the following modifications. First, to make sure that all participants understood the group compositions of the people actually tested in the rule-inadequate trials, the numbers of Xs and Ys in the subsample were explicitly stated (e.g., “in the first day of the study only 5 politicians and 5 nannies were interviewed”), thus overcoming any potential ambiguity of the formulations used in the previous experiments. 

Furthermore, a measure of confidence was added as an additional indicator of conflict detection. After responding to each trial, participants were asked to express the degree to which they were confident in their response. If the opposition between stereotype-based information (traits) and base rates was detected, then the confidence in responses to conflict trials should be lower than the confidence in responses to no-conflict trials (e.g., Bago and De Neys 2017). 

### 5.1. Method

#### 5.1.1. Participants

Sixty-four participants from the University of Lisbon (54 females) performed the experiment in exchange for course credit. Sensitivity analysis with this sample size (at α = .05, and power = .80) showed that the experimental design could reliably detect medium or larger effect sizes (*f ≥* .42).

#### 5.1.2. Material

The same 44 pairs of social groups from the previous experiment were used (translated to Portuguese).

#### 5.1.3. Procedure

The procedure and design were the same as in Experiment 3, except that (a) the distinction between standard and rule-inadequate problems was created by the inclusion of one screen that established whether the target person in the problem was randomly selected from the total sample described in the group composition information screen (“in the first day of the study, all politicians and nannies were interviewed”), or rather from a subset of the sample where base rates are equal (“in the first day of the study only 5 politicians and 5 nannies were interviewed”); (b) after each trial, participants had to indicate how confident they were in their responses on a 9-point rating scale (from 1—not at all confident; to 9—totally confident); and (c) the final CRT task had one extra problem (see Appendix A).

### 5.2. Results

**Figure 6:**
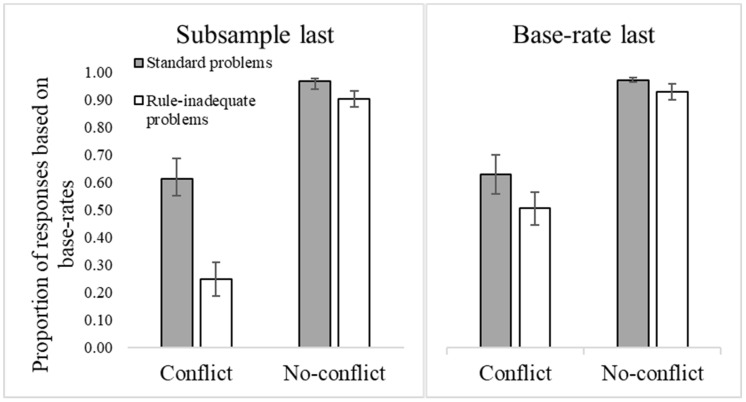
Proportion of correct responses to no-conflict problems and base-rate responses to the standard and rule-inadequate versions of conflict problems in both orders of information presentation (Experiment 4).

Six participants who responded to standard no-conflict problems with accuracies below .80 were removed from the analysis. The mean proportion of base-rate responses for conflict and no-conflict problems for standard and rule-inadequate problems is presented in Figure 6.

#### 5.2.1. Base-Rate Responses

A 2X2X2 ANOVA, with problem Version (standard and rule-inadequate problems) and problem type (conflict and no-conflict) as within-participant factors, order (subsample last; base rate last) as between-participant factor, and mean proportion of base-rate responses as the dependent variable, showed a main effect of version (*F*(1, 56) = 46.91, *p* < .001, η_p_^2^ = .46), such that standard problems led to a higher proportion of base-rate responses (*M* = .79, *SE* = .03) than rule-inadequate problems (*M* = .65, *SE* = .02). There was also a main effect of type, with more responses according to base rates to no-conflict trials (*M* = .94, *SE* = .01) than conflict trials (*M* = .50, *SE* = .04; *F*(1, 58) = 87.98, *p* < .001, η_p_^2^ = .61). Additionally, there was an interaction between version and order (*F*(1, 56) = 9.17, *p* = .004, η_p_^2^ = .14). While the proportion of base-rate responses was lower for rule-inadequate problems (*M* = .58, *SE* = .03) than for standard problems (*M* = .79, *SE* = .04) when the subsample information was presented last (*F*(1, 56) = 9.08, *p* = .004, η_p_^2^ = .14), there were no differences in base-rate responses for standard problems as a function of order (*F* < 1). Furthermore, there was also a version x type interaction (*F*(1, 56) = 34.01, *p* < .001, η_p_^2^ = .38). The difference in proportion of base-rate responses between rule-inadequate and standard problems was significant for both types of problems, but was higher in conflict problems (*M* = .62, *SE* = .05 for standard problems, and *M* = .38, *SE* = .04 for rule-inadequate problems; *F*(1, 56) = 52.22, *p* < .001, η_p_^2^ = .48) than in no-conflict problems (*M* = .97, *SE* = .01 for standard problems and *M* = .92, *SE* = .02 for rule-inadequate problems; *F*(1, 56) = 8.37. *p* = .005, η_p_^2^ = .13). Finally, an interaction between Version, Type, and Order (*F*(1, 56) = 11.37, *p* = .001, η_p_^2^ = .17) showed that the proportion of base-rate responses to conflict rule-inadequate trials was lower when the subsample information was presented last (*F*(1, 56) = 8.81, *p* = .004, η_p_^2^ = .14), but there were no differences in base-rate responses for standard conflict trials (*F* < 1) (see Appendix B, Table A1).

Summing up, the analysis of base-rate responses replicated that of the previous experiments. As in Experiment 3, when the subsample of participants actually tested was the last information presented, participants relied less on irrelevant base rates (in rule-inadequate problems). 

Notably, however, the proportion of base-rate responses to standard and rule-inadequate trials showed a positive and large correlation (*r* = .71, *p* < .001), suggesting an overgeneralization bias (see Table 1). The same positive correlation was observed for participants in the subsample-last condition (*r* = .58, *p* = .001) and in the base-rates last condition (*r* = .86, *p* < .001).

Unfortunately, it was not possible to compare subgroups of high and low cognitive reflection because thirty-three participants (56.90%) erred all CRT problems and only two answered accurately to all problems.

#### 5.2.2. Response Times

RTs (converted to log^10^) were analyzed separately for standard and rule-inadequate problems (see Appendix B, Table A2 for means in ms). 

*Standard Trials.* A 3X2 ANOVA on the mean RT of trials, with response (base-rate response in no-conflict problems, base-rate or stereotype-based in conflict problems) as within-participants factor and order (base rates last, subsamples last) as between-participants factor, yielded a main effect of response (*F*(2, 90) = 7.29, *p* = .001, η_p_^2^ = .14). Responses to no-conflict problems (*M* = 2.99, *SE* = .02) were faster than stereotype-based responses to conflict problems (*M* = 3.14, *SE* = .04; *F*(1, 45) = 21.57, *p* < .001 η_p_^2^ = .32) and were faster than base-rate responses to conflict problems (*M* = 3.05, *SE* = .33), although the difference did not reach statistical significance (*F*(1, 45) = 3.47, *p* = .069, η_p_^2^ = .07).

*Rule-inadequate Trials.* A 3X2 ANOVA on the mean RT of trials, with response (base-rate responses to no-conflict trials, base-rate or stereotype-based responses to conflict trials) as within-participants factor and order (base rates last, subsamples last) as between-participants factor, showed a main effect of response (*F*(2, 92) = 6.17, *p* = .003, η_p_^2^ = .12). Planned comparisons showed that response to no-conflict trials (*M* = 2.97, *SE* = .02) had shorter RTs when compared to base-rate responses to conflict trials (*M* = 3.07, *SE* = .03; *F*(1, 46) = 14.16, *p* < .001, η_p_^2^ = .23), but not when compared to stereotype-based responses to conflict trials (*M* = 3.02, *SE* = .33; *F*(1, 46) = 3.12, *p* = .083, η_p_^2^ = .06). There was also a significant interaction between response and order (*F*(2, 92) = 5.70, *p* = .004, η_p_^2^ = .11). Base-rate responses in conflict trials were significantly slower than responses in no-conflict trials both when subsamples were presented last (*F*(1, 46) = 7.28, *p* = .009, η_p_^2^ = .14), and when base rates were presented last (*F*(1, 46) = 6.89, *p* = .011, η_p_^2^ = .13). Stereotype-based responses in conflict trials, however, were slower than normative responses in no-conflict trials when base rates were presented last (*F*(1, 46) = 11.93, *p* = .001, η_p_^2^ = .21), but not when subsamples were presented last (*F*(1, 46) = 1.10, *p* = .300, η_p_^2^ < .21). 

#### 5.2.3. Confidence

Confidence ratings were analyzed separately for standard and rule-inadequate problems.

*Standard Trials.* A 3X2 ANOVA on confidence ratings of standard trials, with response (base-rate responses to no-conflict problems, base-rate or stereotype-based responses to conflict problems) as within-participant factor and order (base rate last, subsample last) as between-participants factor, showed only a main effect of response (*F*(2, 90) = 13.16, *p* < .001, η_p_^2^ = .23), such that responses to no-conflict trials (*M* = 6.88, *SE* = .17) were given with greater confidence than both base-rate responses (*M* = 6.10, *SE* = .20; *F*(1, 45) = 27.37, *p* < .001, η_p_^2^ = .38) and stereotype-based responses (*M* = 5.78, *SE* = .30; *F*(1, 45) = 22.88, *p* < .001, η_p_^2^ = .34) to conflict trials.

*Rule-inadequate Trials.* A 3X2 ANOVA on confidence ratings, with response (base-rate responses to no-conflict problems, base-rate or stereotype-based responses to conflict problems) as within-participant factor and order (base rate last, subsample last) as between-participants factor, showed a main effect of response (*F*(2, 92) = 11.36, *p* < .001, η_p_^2^ = .20). Responses to no-conflict trials (*M* = 6.74, *SE* = .22) were given with more confidence than both base-rate responses to conflict trials (*M* = 5.82, *SE* = .22; *F*(1, 46) = 25.60, *p* < .001, η_p_^2^ = .36) and stereotype-based responses to conflict trials (*M* = 6.23, *SE* = .26; *F*(1, 46) = 7.92, *p* = .007, η_p_^2^ = .15). There was also a response x order interaction (*F*(2, 92) = 5.50, *p* = .006, η_p_^2^ = .11). When base-rate information was presented last, confidence in correct responses to no-conflict trials was higher than both base-rate responses to conflict trials (*F*(1, 46) = 19.85, *p* < .001, η_p_^2^ = .30) and stereotype-based responses to conflict trials (*F*(1, 46) = 19.46, *p* < .001, η_p_^2^ = .30). However, when subsample information was presented last, confidence in correct responses to no-conflict trials was higher than confidence in base-rate responses to conflict trials (*F*(1, 46) = 7.10, *p* = .011, η_p_^2^ = .13) but did not differ from confidence in stereotype-based responses to conflict problems (*F* < 1).

### 5.3. Discussion

The results pattern replicated that of Experiment 3. Presenting the number of individuals tested after the group composition information and just before participants were prompted to give their response seems to have made this crucial information more salient to the participants, improving their performance in rule-inadequate trials. Importantly, the correlation between the proportion of base-rate responses to standard and rule-inadequate trials observed in Experiments 1 to 3 was even more pronounced in Experiment 4 (in terms of Cohen’s effect sizes, three of the reported correlations were large, and one—Experiment 1—was medium-to-large). 

Response-time and confidence-rating analyses of standard trials provided partial evidence of conflict detection both when participants opted for the stereotype-based response and when they responded according to base rates. A similar pattern of results emerged for rule-inadequate trials.

In sum, these results converge with those of Experiments 1 to 3 and show that participants have difficulties in discriminating between relevant and irrelevant base-rate information. This may lead participants to misuse statistical information and draw conclusions based on irrelevant base rates. 

## 6. General Discussion

In four experiments using two different experimental procedures (moving windows and rapid-response paradigm), we partially replicated the key results of previous research (De Neys and Glumicic 2008; Pennycook et al. 2015). Specifically, we found increased response doubt reflected in terms of longer response latencies, a stronger tendency to revisit the initial base-rate information, and lower confidence ratings (lower feeling of confidence) for responses to conflict versions of standard base-rate problems (when compared to no-conflict problems). This occurred both for base-rate responses and for stereotype-based responses. 

These results support the notion that the opposition, in conflict problems, between stereotype-based and statistical information (i.e., base rates) is detected and possibly triggers more deliberate processing.

Interestingly, the results pattern for rule-inadequate problems mirrored the one just described for standard base-rate problems. Stereotype-based and base-rate responses to conflict versions of rule-inadequate problems (where the base rates opposing the stereotype-based information were irrelevant) also took longer and were given with less confidence than responses to no-conflict versions of the same problems. In other words, participants’ sensitivity to conflict, as reflected by these measures (response times and confidence ratings), does not seem to discriminate a real conflict between stereotype-based and valid base-rate information from a spurious conflict between stereotype-based information and irrelevant base rates. 

This is not to say that participants simply did not distinguish between the two versions of standard and rule-inadequate base-rate problems. In fact, participants responded more often according to base rates when they were a valid source of statistical information (standard problems) than when base rates were irrelevant (rule-inadequate problems). In addition, manipulation of the order of presentation of the information (i.e., presenting the subsample actually used after the base rates and before participants were prompted to respond) further reduced the reliance on base rates on rule-inadequate problems. 

However, a large positive correlation was consistently observed between base-rate responses to standard and rule-inadequate versions of the base-rate problems. This correlation suggests a tendency to use statistical information (base rates) even when it ceases to provide relevant statistical information. This overgeneralization bias is attenuated among high-reflective participants (as measured by the CRT), which is congruent with the notion that inaccurate rule-based reasoning can be a critical source of response bias.

### 6.1. Inadequate Use of Decision Rules in Reasoning about Everyday Problems

Rule-inadequate versions of standard base-rate problems were quite useful in disentangling adequate from inadequate use of base rates in responding to these problems. 

As aforementioned, we are not the first to resort to this type of research strategy. The rationale for our approach was inspired by previous work by Fong et al. (1986). These authors developed so-called false-alarm versions of reasoning problems opposing statistical information (e.g., large but biased samples) to stereotype-based information, to test the effects of brief statistical training on the ability to use statistical principles such as the law of large numbers. By adding these false-alarm problems to their dependent measures, Fong et al. were able to show that statistical training increased the adequate use of the law of large numbers without leading to its over-application to situations where it was not called for.

Klauer and Singmann (2013) used rule-inadequate versions of syllogistic reasoning problems, which they referred to as “pseudo-syllogisms”^7^, to further test Morsanyi and Handley’s (2012) argument that logicality of syllogisms can be detected in an intuitive manner via changes in affective state (conceptual fluency). They showed that the surface features of the problems were confounded with their logical status and that these features, rather than an implicit intuitive access to the logically correct solution, drove participants’ responses. 

Likewise, the rule-inadequate versions of the base-rate problems used in the research here reported make the base-rate information irrelevant for the judgment, while leaving the surface features of the problems largely unchanged (e.g., information concerning group composition is still presented). In this sense, rule-inadequate problems may be useful for determining whether conflict-detection effects are driven by surface features that happen to covary with the normative implications of the problems (i.e., the spurious conflict between invalid base rates and stereotype-based information), or whether the normative implications themselves are causally responsible.

Analogous to the work of Klauer and Singmann (2013) in syllogistic reasoning, the use of what we referred to as rule-inadequate problem version (i.e., false-alarm problems) revealed that measures of conflict detection do not necessarily show participants’ sensitivity to normative prescriptions. This may contribute to a better characterization of the interaction between different T1 intuitions and of T2 thinking.

### 6.2. Contributions to the Debate among Dual-Process Models of Reasoning

Two prevalent notions in the dual-process literature of reasoning are (a) the view that T2 thinking is responsible for the application of relevant rules of logic and probability that often correct T1 outputs (e.g., Kahneman 2003); and (b) that even when opting for the heuristic-based response in classic reasoning tasks, people have competing logical intuitions (i.e., intuitive access to logical and probabilistic principles) that point to the normatively correct response in classic reasoning tasks, such as base-rate problems (e.g., De Neys 2012). Previous research (Klauer and Singmann 2013) and our results suggest that both of these notions may need to be qualified. 

Indeed, the present findings suggest that a sizeable fraction of those individuals who appear to respond in an adequate fashion (i.e., according to the normative implications of base rates) in standard base-rate problems may often also rely on statistically irrelevant information rather than making an adequate use of formal rules. In the same vein, processing measures such as (longer) response times and (decreased) confidence may point to the existence of faulty logical intuitions (opposing heuristic ones). This might occur if logical intuitions are at least sometimes driven by salient surface features (e.g., extreme base rates opposing stereotype-based information) that happen to co-vary with sound probabilistic judgment in the case of standard base-rate problems but not in the case of rule-inadequate versions of these problems.

In other words, what have been termed logical intuitions (e.g., De Neys 2012) may not always follow valid probabilistic or logic principles. These intuitions may sometimes merely conform to this principle in standard base-rate problems (though, to be sure, some studies offer compelling evidence for logical intuitions—e.g., Frey et al. 2018; Šrol and De Neys 2021). 

Inferring the sound use of statistical rules from normatively correct responses to standard conflict problems might therefore be unwarranted when the tendency to use base rates, even when they are not a relevant source of statistical information (overgeneralization bias), is not considered. 

Further research is certainly needed to clarify the conceptual implications of the current findings. The two-response procedure developed and validated by Thompson et al. (2011) could be used to better examine the time course of intuitive and deliberate processing in rule-inadequate problems. In this procedure, participants are asked to give two consecutive responses. At time 1, they are encouraged to respond intuitively and are put under a deadline to minimize the role of analytic thinking. They then provide feeling of rightness (FOR), followed by a second answer, during which reflection is encouraged. Two measures of T2 engagement are obtained: the length of the rethinking period and the probability of changing the initial answer. To what extent faulty logical intuitions would emerge at time 1, and to what extent associated FOR would predict T2 engagement and the confirmation or correction of the initial response to rule-inadequate problems, are interesting research questions waiting to be explored.

In conclusion, the present findings add to the growing body of evidence that challenges the traditional dual-process perspective (e.g., De Neys 2012; De Neys and Pennycook 2019; Newman et al. 2017; Thompson et al. 2018). However, they also beg for a more nuanced interpretation of findings suggesting that people have intuitive access to accurate logic and probability principles (e.g., De Neys 2012; De Neys and Pennycook 2019). It may be the case that sometimes people have intuitive access to faulty logical principles, which may eventually lead to biased rule-based responses. 

## Figures and Tables

**Table 1:** Correlation of base-rate responses between standard and rule-inadequate problems (Experiments 1 to 4).

	*r (p)*	High Reflective *r (p)*	Low Reflective *r (p)*
Experiment 1	.46 (<.001)		
Experiment 2	.50 (<.001)	.05 (.773)	.70 (<.001)
Experiment 3	.53 (<.001)	.32 (.138)	.65 (<.001)
Experiment 4	.71 (<.001)		

**Table 2:** Evidence of conflict detection for standard and rule-inadequate problems (Experiments 1 to 4).

	Response Time Higher than Response Time for Problems without Conflict		Confidence in Responses Lower than for Responses to Problems without Conflict (only Experiment 4)		Base-Rate Reviewing Time (only Experiment 1)	
	Responses Based on Base Rate	Responses Based on Stereotypes	Responses Based on Base Rate	Responses Based on Stereotypes	Responses Based on Base Rate	Responses Based on Stereotypes
Experiment 1						
Standard	X	X				
Rule-inadequate					X	
Experiment 2						
Standard	X	X				
Rule-inadequate	X	X				
Experiment 3						
Standard	X	X				
Rule-inadequate	X	X				
Experiment 4						
Standard	X	X	X	X		
Rule-inadequate	X	*	X	X		

* Stereotype-based responses were slower than responses to no-conflict trials only when information regarding base rates was presented last.

**Table 3:** Information displayed in conflict/no-conflict and standard/rule-inadequate problems in the base-rate neglect tasks of the present studies.

	For a Person Randomly Selected from the Sample with the Trait “Kind”:	
	Base Rate of the Groups in the Study	Base Rate from Which a Person Was Randomly Selected
Standard conflict	995 politicians and 5 nannies	All politicians and nannies
Rule-inadequate conflict	995 politicians and 5 nannies	An equal sample of politicians and nannies/5 politicians and 5 nannies *
Standard no-conflict	5 politicians and 995 nannies	All politicians and nannies
Rule-inadequate no-conflict	5 politicians and 995 nannies	An equal sample of politicians and nannies/5 politicians and 5 nannies *

* Format used in Experiment 4.

**Table 4:** Means and standard deviations of base-rate responses for different types and versions of problems for low- and high-reflective reasoners in Experiments 2 and 3.

		Low-Reflective Reasoners (*N* = 70)		High-Reflective Reasoners (*N* = 43)	
Type of Problem	Version of Problem	*M*	*SD*	*M*	*SD*
Experiment 2					
No-conflict	Standard	.98	.04	.98	.03
	Rule-inadequate	.94	.10	.97	.07
Conflict	Standard	.68	.29	.84	.20
	Rule-inadequate	.49	.32	.54	.31
Difference between conflict problems		.19		.30	
Experiment 3					
No-conflict	Standard	.97	.06	.98	.07
	Rule-inadequate	.95	.08	.94	.10
Conflict	Standard	.60	.33	.74	.27
	Rule-inadequate	.34	.32	.23	.25
Difference between conflict problems		.26		.51	

## Data Availability

The data presented in this study are available on request from the corresponding authors.

## Notes

1 The base rates presented in the original version of the lawyer–engineer problem (70 lawyers and 30 engineers; Kahneman and Tversky 1972) were replaced in this example for more extreme base rates in order to make sure that when the base rates are taken into account, the likelier response option is “Dan is a lawyer”).

2 Base rates and group composition coincide in the case of standard problems but not in the rule-inadequate problems, where base rates are 50/50. However, for ease of presentation, throughout the text we refer to “base-rate responses” for both standard and rule-inadequate problems. These are adequate responses in the case of standard problems and responses based on invalid base rates in the case of rule-inadequate problems.

3 The number of participants in Experiment 1 and in the remaining experiments here reported were not pre-defined based on power analysis. Experiments were included in different experimental waves, which were ran to completion. The obtained Ns are comparable to previous studies using the same experimental paradigms. Specifically, a total of 86 participants completed De Neys and Glumicic’s (2008) Experiment 2 (the same number completed our Experiment 1). The number of participants who completed Pennycook et al.’s (2015) Studies varied between N = 60 and N = 88; whereas in the Experiments 2 to 4 here reported, they varied between N = 64 and N = 116). Tests of sensitivity were ran for all studies.

4 By “correct responses to no-conflict problems” we mean responses according to the base rates and stereotype-based information, which both converge in the same response option in these problems.

5 We thank Gordon Pennycook for making available to us the sample of traits used in the studies in Pennycook et al. (2015).

6 The asymmetry in the number of standard problems—twice as many as the rule-inadequate problems—was a mistake in the code of the E-prime program used to run this Experiment. In Experiment 3, this issue was corrected.

7 “pseudo-syllogisms” are false alarm versions of classical syllogisms in the sense that they modify the logical (valid or invalid) status of the syllogism while keeping the superficial structure of the problems unchanged.

## Appendix A

Problems from the cognitive reflection test presented in the experiments

In Experiment 2:

1- If 3 workers need 3 min to produce 3 toys, how long would it take 500 workers to produce 500 toys?

2- A TV and a DVD player cost, together, 110€. The TV costs 100€ more than the DVD player. How much costs the DVD player?

3- A virus is spreading in a computer. Every minute, the number of infected files double. If it takes 100 min to infect the whole system, how long does it take to infect half of the system?

Added in Experiment 4:

4- John had both the 15° best note and the 15° worse note on the class exam. How many students, in total, has the class?

## Appendix B

**Table A1:** Means and standard errors of base-rate responses for different problems in each experiment.

	Subsample Last	Base Rates Last
	*M (SE)*	*M (SE)*
Experiment 1		
Standard no-conflict	.97 (.03)	-
Standard conflict	.46 (.03)	-
Rule-inadequate conflict	.26 (.03)	-
Experiment 2		
Standard no-conflict	-	.98 (<.01)
Standard conflict	-	.74 (.03)
Rule-inadequate no-conflict	-	.95 (.01)
Rule-inadequate conflict	-	.51 (.03)
Experiment 3		
Standard no-conflict	.95 (.01)	.98 (.01)
Standard conflict	.64 (.05)	.64 (.05)
Rule-inadequate no-conflict	.93 (.01)	.96 (.01)
Rule-inadequate conflict	.23 (.04)	.40 (.05)
Experiment 4		
Standard no-conflict	.97 (.01)	.97 (.01)
Standard conflict	.61 (.07)	.63 (.07)
Rule-inadequate no-conflict	.90 (.03)	.93 (.03)
Rule-inadequate conflict	.25 (.06)	.50 (.06)

**Table A2:** Response times (in ms) for each kind of response in each type of problem across experiments.

	No Conflict	Conflict		
	Correct	Base Rate	Stereotype	
	*M (SE)*	*M (SE)*	*M (SE)*	N Included in the Analysis
Experiment 1				
Standard	9615.04 (839.41) *	12,386.22 (839.41)	12,052.92 (839.41)	44
Rule-inadequate	10,117.63 (857.88) *	11,995.65 (857.88)	10,987.56 (857.88)	34
Experiment 2				
Standard	1045.85 (103.76)	1390.52 (103.76)	1847.12 (103.76)	84
Rule-inadequate	1037.08 (201.68)	2072.12 (201.68)	1229.29 (201.68)	94
Experiment 3				
Standard	1016.19 (99.99)	1199.60 (99.99)	1830.12 (99.99)	70
Rule-inadequate	1056.20 (90.83)	1584.46 (90.83)	1227.47 (90.83)	72
Experiment 4				
Standard	1051.35 (114.95)	1292.32 (114.95)	1651.77 (114.95)	47
Rule-inadequate	1007.27 (99.06)	1349.53 (99.06)	1188.64 (99.06)	48

* The response time associated to Rule-inadequate problems in Experiment 1 is from no-conflict standard problems, because there was no Rule-inadequate versions of no-conflict problems in this experiment. Because of different number of missed responses in standard and rule-inadequate conflict trials, different means were taken in account in each ANOVA.

**Table A3:** Examples for all variations of problems used in Experiment 1.

	Problem Type	
Problem Version	No-Conflict	Conflict
Standard problems	In a study 1000 people were tested. Among the participants there were 4 women and 996 men. *In the first week of the study, all men and women were interviewed. Jo was one of the people interviewed in the first week.* Jo is 23 years old and is finishing a degree in engineering. On Friday nights, Jo likes to go out cruising with friends while listening to loud music and drinking beer. What is most likely? a. Jo is a man b. Jo is a woman	In a study 1000 people were tested. Among the participants there were 996 women and 4 men. *In the first week of the study, all men and women were interviewed. Jo was one of the people interviewed in the first week.* Jo is 23 years old and is finishing a degree in engineering. On Friday nights, Jo likes to go out cruising with friends while listening to loud music and drinking beer. What is most likely? a. Jo is a man b. Jo is a woman
Rule inadequate		In a study 1000 people were tested. Among the participants there were 996 women and 4 men. *In the first week of the study, as many men as women were interviewed. Jo was one of the people interviewed in the first week.* Jo is 23 years old and is finishing a degree in engineering. On Friday nights, Jo likes to go out cruising with friends while listening to loud music and drinking beer. What is most likely? a. Jo is a man b. Jo is a woman
